# Enhanced Bioremediation Potential of *Shewanella decolorationis* RNA Polymerase Mutants and Evidence for Novel Azo Dye Biodegradation Pathways

**DOI:** 10.3389/fmicb.2022.843807

**Published:** 2022-03-22

**Authors:** Xunchao Cai, Xin Zheng, Yicheng Wang, Li Tian, Yanping Mao

**Affiliations:** ^1^College of Chemistry and Environmental Engineering, Shenzhen University, Shenzhen, China; ^2^Department of Gastroenterology and Hepatology, Shenzhen University General Hospital, Shenzhen, China

**Keywords:** *Shewanella decolorationis*, RNA polymerase mutation, azo dye degradation, co-expression network, bioremediation

## Abstract

Bioremediation has been considered as a promising method for recovering chemical polluted environments. Here *Shewanella decolorationis* strain Ni1-3 showed versatile abilities in bioremediation. To improve the bioremediation activity, RNA polymerase (RNAP) mutations of strain Ni1-3 were screened. Eleven mutants were obtained, of which mutant #40 showed enhanced Amaranth (AMR) degradation capacity, while mutant #21 showed defected capacity in AMR degradation but greatly enhanced capacity in cathodic metal leaching which is three to four times faster than that of the wild-type (WT) strain Ni1-3, suggesting that different pathways were involved in these two processes. Transcriptional profiling and gene co-expression networks between the mutants (i.e., #40 and #22) and the WT strain disclosed that the non-CymA-Mtr but cytochrome *b*- and flavin-oxidoreductase-dominated azo dye degradation pathways existed in *S. decolorationis*, which involved key proteins TorC, TorA, YceJ, YceI, Sye4, etc. Furthermore, the involvement of TorA was verified by trimethylamine N-oxide reduction and molybdenum enzyme inhibitory experiments. This study clearly demonstrates that RNAP mutations are effective to screen active microbial candidates in bioremediation. Meanwhile, by clarifying the novel gene co-expression network of extracellular electron transfer pathways, this study provides new insights in azo dye degradation and broadens the application of *Shewanella* spp. in bioremediation as well.

## Introduction

Human civilization has been greatly accelerated since the industrial revolution. However, side effects of releasing toxic chemicals to water, land, and air brought by anthropogenic activities have become a major source of worldwide environmental pollution (Larsson, 2014; Cheng, 2016; Dangi et al., 2019). The most concerned pollutants include synthetic dyes, heavy metals, pesticides, and so on (Deblonde et al., 2011; Xu et al., 2016). Among these pollutants, synthetic dyes and e-wastes have been recognized as highly toxic and hazardous members by major environmental agencies and organizations (Vikrant et al., 2018; Filomeno and Feraco, 2020).

Azo dyes account for about 70% of the world’s synthetic dyestuff production, which are extensively used in the textile, leather, food, and many other industries (Saratale et al., 2011). The discharge of azo dye effluvium would cause serious environmental pollutions and pose lethal effects, genotoxicity, or mutagenicity to creatures (Puvaneswari et al., 2006; Zhao et al., 2011; Qiu et al., 2014; Chung, 2016; Ajaz et al., 2019). However, azo dyes are validated as one of the persistent contaminants in wastewater treatment processes (Venkata et al., 2005; Gupta and Gupta, 2016), requiring great efforts to get them removed. In recent years, the rapid development of electronic industries produces increasing number of e-wastes, such as spent lithium-ion batteries (LIBs) (Filomeno and Feraco, 2020). The expanding LIB market produces large amounts of spent LIBs that highly contain toxic metals and increases the demand for metal sources contained in LIBs (Zeng et al., 2014; Boxall et al., 2018). Recycling metals from spent LIBs cannot only protect the environment but also save natural resources, thus maintaining the sustainable development of electronic industries (Barik et al., 2017). Although several physical and chemical approaches have been reported to tackle the azo dye or e-waste pollution in the environment (Chen T. et al., 2010; Naaz et al., 2017), they require large quantities of chemicals or energy. Besides, byproducts cannot be fully removed, and secondary pollution often occurred due to the large amount of surplus sludge or toxic gas (Bahaloo-Horeh et al., 2018; Mani et al., 2019). In contrast, bioremediation is considered as an eco-friendly, cost-effective, and easy-operating way to eliminate these pollutants from the environment (Popli and Patel, 2014). However, bioremediation takes a much longer time and disposes much lower concentrations of pollutants when compared to the other methods (Liu et al., 2013a), the further application of bioremediation in azo dye degradation and spent LIB disposal using bacteria faces bottlenecks to be overcome.

*Shewanella* spp. are well known for their versatile electron-donating abilities, which have considerable potential in the remediation and detoxification of various contaminants such as oxidized metals, nitrate, trimethylamine *N*-oxide (TMAO), and azo dyes (Luan et al., 2009; Burns et al., 2010; Cai et al., 2011). More importantly, *Shewanella* strains often thrive under conditions with high salinity as well as heavy metal pressure in the environments (Khalid et al., 2008; Meng et al., 2012), which is one of the biological benefits for them to be used for azo dye removal in practice. Although there is little evidence showing the bioleaching capacity of *Shewanella* spp. on spent LIBs, the reduction capacity of *Shewanella* spp. on solid oxidized metals [e.g., Mn(IV) and Fe(III)] have been observed (Cai et al., 2011). We have noticed that the metals in spent LIBs are in their oxidized status [e.g., Ni(IV), Co(IV), and Mn(IV)], which might be reduced by *Shewanella* spp. and be released into the culture solution from the cathodic materials.

In order to screen out *Shewanella* strains with high bioremediation capacity (e.g., reduced processing time and enhanced disposal quantity) on degrading azo dye or bioleaching spent LIBs, unbiased RNA polymerase (RNAP) mutation screening was applied in this study, which has been proven effective in making alterations in pleiotropic phenotypes and global transcription profiles (Cai et al., 2017). In detail, a wild-type (WT) strain *S. decolorationis* Ni1-3 (hereafter referred to Ni1-3) showing multiple heavy metal resistance (Cai et al., 2019) and high capabilities in azo dye degradation and metal reduction was used as a parental strain to screen spontaneous RNAP mutations, thus obtaining mutants with enhanced bioremediation capacity. Meanwhile, RNA-seq and comprehensive bioinformatic analysis combined with phenotype validation experiments were applied to elucidate the correlations among electron transfer pathways in Ni1-3 and to clarify the mechanisms behind the high azo dye degradation activity.

## Materials and Methods

### Bacterial Strains and Growth Conditions

The WT strain *S. decolorationis* Ni1-3 (deposit no. CGMCC 1.13809) was isolated from the activated sludge of an electroplating sewage treatment plant (Dongguan, China) (Cai et al., 2019). Bacterial strains used in this study were routinely cultured under aerobic conditions on Luria-Bertani (LB) medium, which contained 5 g/L yeast extract, 10 g/L tryptone, and 10 g/L NaCl at 37°C with shaking at 180 rpm. Agar powder was added into the LB medium at a proportion of 2% (m/m) when preparing the LB plate.

### Screening and Characterizing the RNA Polymerase Mutations

The frozen WT strain at −80°C was thawed to inoculate into the LB medium at the proportion of 10% (v/v) and routinely cultured as described above for about 12 h. After that, the cell culture was spread on LB agar plates containing 50 μg/ml rifampicin, which were then incubated at 37°C for 24 h to screen spontaneous RNAP (*rpoB*) mutants showing rifampicin resistance (Rif^r^) (Cai et al., 2017). Colonies appearing on the Rif^r^ plate were picked up sterilely and cultured separately in the LB medium for multiplication and stored at −80°C. The *rpoB* gene amplicon of each strain was obtained using the PCR method (see Supplementary Materials and Methods section “PCR of the *rpoB* Gene”). Amplicons were sequenced by the BGI Company (Shenzhen, China), and each amplicon sequence was aligned against the *rpoB* sequence of WT strain by using the BioEdit software to identify the mutation (Hall, 1999). After that, the minimum inhibitory concentrations (MICs) to rifampicin of each mutant and the WT strain were measured according to the previously described method (Cai et al., 2017). Ten-microliter overnight culture of each strain was inoculated into a sterilized 96-well plate with 190 μl LB medium. The concentration of rifampicin was identified as the MIC when no visible bacterial growth was observed after 24 h incubation at 37°C.

### AMR Degradation and Spent Lithium-Ion Battery Bioleaching

AMR (AR, Solarbio, Beijing, China) used in the study was a typical azo dye which was highly polarized by sulfate groups (Supplementary Figure 1). The anaerobic degradation of AMR was carried out in the modified MR2A medium (Fries et al., 1994). Meanwhile, KCl was substituted for KNO_3_ to avoid the interference of nitrate as an electron acceptor (Liu et al., 2018). Lactate (20 mM) and AMR (8 mM) were added as the electron donor and acceptor, respectively. The monitoring and measurement of AMR biodegradation were done through an efficient high-throughput assay system at 37°C (Xiao et al., 2018), which contained 190 μl mixed solution (AMR and medium), 10 μl bacterial seed inoculum (see Supplementary Materials and Methods section “Seed Inoculum Preparation and Bacerial Growth Monitoring”), and 50 μl petrolatum oil. The concentration of AMR in the 96-well plate was calculated by measuring the optical density at 520 nm with a microplate reader (Synergy HTX Multi-Mode Reader, BioTek, United States) using the standard curve method to achieve real-time and non-destructive monitoring. All of the experiments described above were conducted in triplicate. The degradation efficiency was calculated according to the described method (Xiao et al., 2018). The cathodic metal leaching capacity was determined by calculating leaching efficiency of the cathodic material NCM523 that consisted of oxidized Ni, Co, and Mn. The original contents of unleached Ni, Co, and Mn material in liquid mineral salt medium (MSM) (Imran et al., 2016) were equal to 4.94, 1.98, and 3.18 mM, respectively, and 10% (vol/vol) seed inoculum was inoculated into MSM (50 ml serum bottles filled with 25 ml MSM). Then, nitrogen gas was used to purge the bottles for 5 min to remove the residual oxygen, and the serum bottles were sealed with butyl rubber stoppers. Metal concentration in the medium was measured using SQ-ICP-MS (Thermo Fisher Scientific iCAP RQ ICP-MS, United States), and the leaching efficiency was calculated according to Bahaloo-Horeh et al.’s (2016) method after static incubation at 37°C for 48 h.

### Sampling and RNA-Seq

The WT strain and two RNAP mutants with either the highest or lowest AMR degradation efficiency compared to WT strain were separately inoculated into MR2A (20 mM lactate and 8 mM AMR added) in 50 ml serum bottles (25 ml medium per bottle), and then nitrogen gas was used to purge the bottles for 5 min to remove residual oxygen. Then, 5 ml petrolatum oil was carefully added to the surface of the mixed culture, and the serum bottles were sealed with butyl rubber stoppers. The prepared serum bottles with cultures were then incubated constantly at 37°C. One milliliter culture was collected using RNase-free pipette in a 30-min interval for AMR degradation efficiency determination or RNA-seq sampling. Cell pellets were harvested by centrifuged at 4°C, 12,000 × *g* for 1 min and then immediately put into liquid nitrogen for 5 min and transferred to −80°C refrigerator for storage. The supernatant was filtrated using a 0.45 μm filter to remove the remaining bacterial cells, which was then used for AMR measurement. The samples at two time points of each strain with significant degradation efficiency difference were selected for RNA-seq sampling. Each treatment was biologically prepared with triplicate. The selected samples were transferred into drikold package and sent to the Novogene Company (Nanjing, China) for RNA extraction and RNA-seq (see Supplementary Materials and Methods section “RNA-Seq”).

### Data Processing and Differential Expression Analysis

TrimGalore (v0.6.6)^1^ was used for the quality control of clean reads obtained from the Novogene Company. The reference genome (accession no. CP031775) was downloaded from the genome database of NCBI, and the genome index was built using HISAT (v2.2.1) (Kim et al., 2015). Then the quality-controlled reads were mapped to the index file and reference gff3 file to produce bam files using HISAT (v2.2.1) and samtools (v1.11)^2^. Gene counts of each sample were calculated from the bam file using HTSeq (v0.11.3)^3^. To check the biological replication of each sample, pairwise scatter plot of the raw count data and multidimensional scaling (MDS) plot of the normalized count data were conducted using the R package edgeR (Robinson et al., 2010). Generalized linear models implanted in edgeR were then used to calculate the differentially expressed genes (DEGs) between the mutants and the WT strain. Genes with log2(fold change) ≥ 1 or log2(fold change) ≤ -1 together with *p* ≤ 0.05 were identified as DEGs. Besides, the relative expression level of each gene was represented using transcripts per million (TPM), which was calculated using salmon (v1.3.0) (Patro et al., 2017). Moreover, a comprehensive gene annotation was conducted using eggNOGmapper5.0^4^.

### Gene Co-expression Network Construction

Weighted gene co-expression network analysis (WGCNA) was then conducted using R software package WGCNA (Langfelder and Horvath, 2008) to find highly correlated genes on the anaerobic AMR degradation process. The TPM data sets containing all the samples were first clustered by hierarchy clustering in WGCNA to check if there were any outliers that should be removed. Then, the soft power used to construct a scale-free network was determined when the model fitting index *R*^2^ = 0.9. Finally, a dynamic tree-cut method was used to identify the co-expression gene modules of the whole transcriptome (minModuleSize = 30 and mergeCutHeight = 0.25). The module network files were then exported and characterized as well as visualized using Cytoscape (v3.7.1) (Shannon et al., 2003). Two methods were combined to extract hub genes in each module. One was based on network connectivity (i.e., node degrees), which extracted genes with the top 5% highest connectivity in the network (Yang et al., 2014). The other was based on module membership (MM) and gene significance (GS), which extracted genes with an MM absolute value of top 10% and an absolute value of GS higher than 0.8, modified according to the method of Tang et al. (2018). Then, the hub genes identified by both methods were considered as hub genes of the networks, and key genes involved in the biological trait were mined from hub genes through database searching and reference exploring. Furthermore, the interactions between hub gene encoded proteins were explored using STRING^5^, selecting the model strain *Shewanella oneidensis* MR-1 as the reference.

## Results and Discussion

### Characterization of the RNA Polymerase Mutations in *Shewanella decolorationis* Ni1-3

**FIGURE 1:**
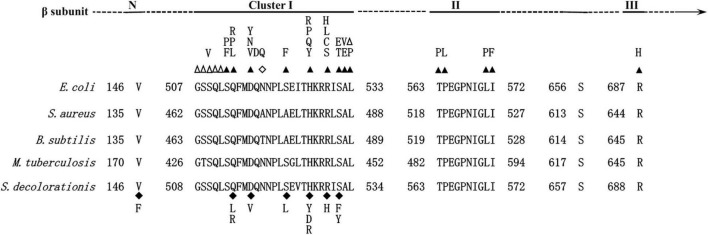
Map of the *S. decolorationis* RNAP β (RpoB) subunit. **(Top)** Location of Rif^r^ clusters I, II, and III. **(Bottom)** Amino acid sequence alignment of the Rif^r^ clusters between *E. coli*, *Staphylococcus aureus*, *Bacillus subtilis*, *Mycobacterium tuberculosis*, and *S. decolorationis*. The numbering begins from the first amino acid of the RpoB sequences. Well-characterized RpoB substitutions that cause rifampicin resistance in *E. coli* are noted above the *E. coli* RpoB sequence. Closed triangles above the *E. coli* sequence indicate amino acid substitutions; empty triangles indicate amino acid deletions; empty diamonds indicate amino acid insertions; closed diamonds below the *S. decolorationis* sequence indicate amino acid substitutions in the *S. decolorationis* RpoB sequence.

To obtain mutants with enhanced bioremediation potential, spontaneous RNA polymerase mutations were screened. After 24 h culturing of WT strain under rifampicin pressure, a total of 47 RNAP mutants with mutations in the β subunit appeared on the Rif^r^ LB plate. Finally, 11 unique RNAP mutants were obtained, and all of them were single-point mutations which resulted in an amino acid (AA) substitution in RpoB (Supplementary Table 1). Among them, AA mutations in the position of 146, 532, and 527 were most frequently observed, taking proportions of 19.15, 25.53, and 23.40%, respectively (Supplementary Table 1), suggesting that there are some hot mutation spots on the RpoB. It has been reported that different sites of the RpoB have different frequencies to mutate. For instance, H526 and S531 in *E. coli* proved to mutate most frequently (Alifano et al., 2015), which were the homologous AA sites to H527 and S532 of Ni1-3, respectively. As shown in Figure 1, the RpoB sequence of Ni1-3 is highly homologous to *E. coli* and other bacterial species. It was found that 10 out of the 11 mutations in Ni1-3 were mapped to a rather restricted region within Cluster I of RpoB (Figure 1). Besides, V146 presented outside of the hot mutation clusters of RpoB, and it was a novel mutation site found in recent years (Alifano et al., 2015). Notably, S523L in this study presented as a novel AA substitution mutation in Cluster I of RpoB (Figure 1). However, no mutations in Cluster II or Cluster III have been observed in Ni1-3. More efforts are needed if we intend to isolate mutants carrying AA mutations in Clusters II and III from Ni1-3, which may increase the possibility to obtain mutants desired for bioremediation.

### Bioremediation Phenotype of the RNA Polymerase Mutants

**FIGURE 2:**
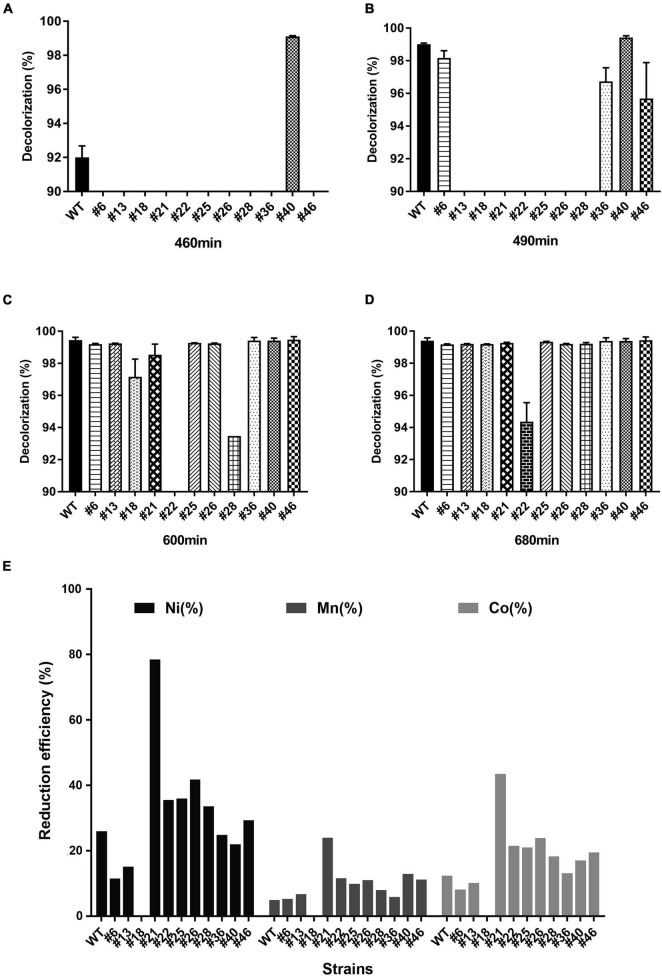
Anaerobic degradation of AMR and bioleaching of cathodic materials by *S. decolorationis* Ni1-3 and RNAP mutants. The initial concentration of AMR was 8 mM. Incubation conducted anaerobically at 37°C after 460 min **(A)**, 490 min **(B)**, 600 min **(C)**, and 680 min **(D)**. The cathodic material NCM523 was from the spent LIBs, and the measurement was conducted after 48 h of incubation **(E)**.

After characterizing the RNAP mutants, we further measured the growth curve (see Supplementary Materials and Methods section “Seed Inoculum Preparation and Bacerial Growth Monitoring”), the azo dye degradation activity, and the bioleaching activity of Ni1-3 and the mutants to assess their potential in bioremediation. The growth curves showed that growth alternations appeared clearly after the late stage of the logarithmic phase (Supplementary Figure 2). Mutant #40 showed the highest degradation activity as AMR was completely degraded at 460 min (Figure 2A). Conversely, other mutants, especially #13, #18, #21, #22, #25, #26, and #28, showed the defected AMR degradation activity (Figures 2B,C), of which #22 showed the lowest activity as AMR cannot be completely degraded after 680 min of incubation (Figure 2D). Consequently, mutant #40 could be designated as a positive mutant for AMR degradation, while seven others (i.e., #13, #18, #21, #22, #25, #26, and #28) as negative mutants. Compared to previous studies, Ni1-3 and most of the mutants showed excellent capacities to degrade AMR, as the concentration of AMR in those studies was 0.1–1 mM, which took 6–40 h for complete degradation (Hong et al., 2007; Chen X. J. et al., 2010; Liu et al., 2013b), and complete removal of 2 mM AMR in *Shewanella algae* and *Shewanella marisflavi* took about 34 and 52 h, respectively (Liu et al., 2013a). As for the most studied *S. decolorationis* S12, to our knowledge, the complete removal of 7 mM AMR in microbial fuel cells (MFCs) and anaerobic reactors took 24 and 20 h, respectively (Yang et al., 2015). Notably, Ni1-3 and the mutants in this study were also competent to degrade AMR effectively under aerobic conditions, promoting the feasibility for these strains to be applied in practice (Supplementary Figure 3).

Importantly, bioleaching capacities to spent LIBs’ cathodic materials (NCM523) were observed being carried by Ni1-3 and the mutants, although the leaching efficiency showed a different pattern as that of AMR degradation (Figure 2E). Mutant #18 presented a great deficiency in bioleaching, while mutant #21 showed greatly enhanced bioleaching capacity with a leaching efficiency of Ni, Mn, and Co in 48 h at 78.43, 23.86, and 43.39%, respectively. Moreover, the negative mutant #22 showed higher leaching capacity than Ni1-3 (Figure 2E), suggesting that the divergent extracellular electron transfer (EET) pathway may be involved between the AMR degradation and the cathodic material leaching. Practically speaking, using mutant #21 for cathodic material bioleaching would be a better way than the traditional bioleaching methods by using obligate and acidophilic chemoautotrophs mostly (Ban et al., 2013), which would greatly save the operation time and capital costs, because the latter method may take 12–14 days to get only 30% Ni and 40% Co leached out (Xin et al., 2016). These results suggested that Ni1-3 and the mutants could be promising candidates for bioremediation in industrial scale. Furthermore, it is reliable to obtain bacterial mutants with high efficiency in biodegrading azo dyes or bioleaching cathodic materials of spent LIBs through screening the RNAP mutants.

### Overview of Gene Transcriptional Profiles Between the Mutants and Wild-Type Strain

**FIGURE 3:**
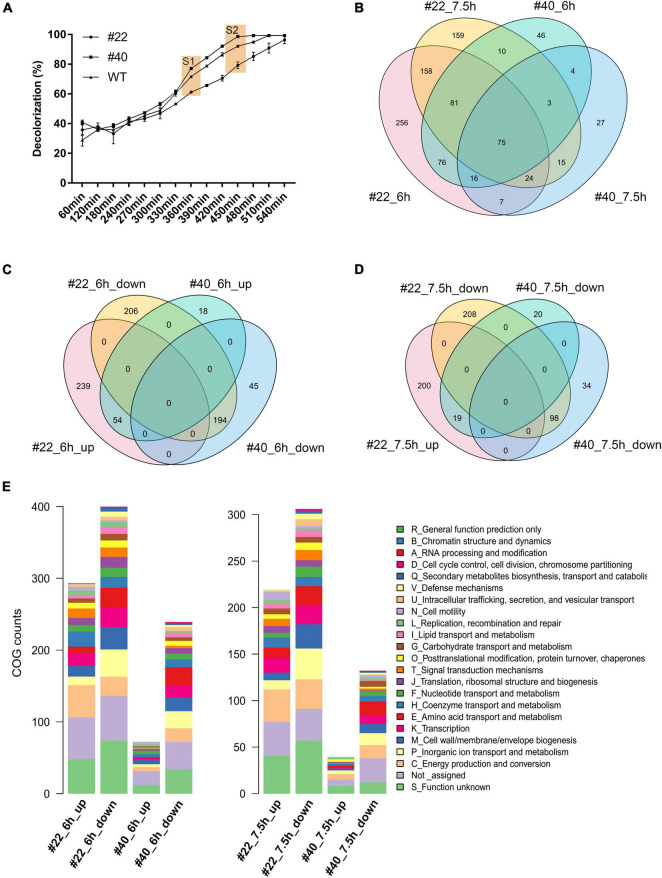
The sampling and profile of the alternation of global gene transcription between strains. S1 and S2 represent the first and second sampling time points, respectively **(A)**. Venn diagram of DEGs between different strains at different time points **(B–D)**. COG function of the DEGs in the mutants at different sampling time points **(E)**.

To explore the transcriptional profile alterations brought by RNAP mutations, RNAP mutants showing the highest AMR degradation activity (i.e., #40) and the lowest AMR degradation activity (i.e., #22) together with the WT strain were selected to conduct RNA-seq. By monitoring the AMR degradation curve in the serum bottles, two time points were selected for RNA-seq analysis. The first time point was 6 h after inoculation when 61.22–78.12% of the AMR was degraded in three strains (i.e., #22_6h, 61.22%; WT_6h, 72.74%; and #40_6h, 78.12%). The second time point was 7.5 h after inoculation when AMR was completely degraded by the positive mutant #40 (Figure 3A). Pairwise scatter plot of the count data showed that one of the biological replicates of #22 (i.e., #22_6h_2) was significantly divergent from the others, which should be removed as an outlier at the DEG calculation process (Supplementary Figure 4A). Multidimensional scaling (MDS) analysis then showed that the remaining samples with the same treatments could be clustered together (Supplementary Figure 4B). Overall, 293 and 72 genes were upregulated, while 400 and 239 genes were downregulated at 6 h in #22 and #40, respectively. Then, 219 and 39 genes were upregulated, while 306 and 132 genes were downregulated at 7.5 h in #22 and #40, respectively (Figures 3B–D). It was observed that more DEGs were downregulated genes in the mutants across the sampling duration, especially for #22 at 7.5 h, implying that RNAP mutations globally affected the transcriptional profiles and resulted in a fitness loss to the WT strains, which reflected in the growth curve as slower growth (Supplementary Figure 2). A similar fitness loss has also been observed in the RNAP mutations in *E. coli*, which showed slow growth and cold- or temperature-sensitive phenotypes (Jin et al., 1988), and a server transcription termination deficiency was observed in both *E. coli* (H526Y) (i.e., H527 in *S. decolorationis* Ni1-3) and *B. subtilis* (H482Y) (Jin et al., 1988; Heisler et al., 1996; Ingham and Furneaux, 2001). Unexpectedly, no contradicting gene regulation pattern was observed between the mutants. For instance, the upregulated genes in #22 were not downregulated in #40, and *vice versa* (Figures 3C,D), suggesting that mutations H527R and D517V may affect the global gene transcription in a similar way as they lay closely in the RpoB sequence (Figure 1) or in the three-dimensional structure (i.e., the rifamycin ansa-moiety) of the β subunit of RNAP. COG annotation of DEGs showed that RNAP mutations resulted in significant alterations in functional profiles. Although a large proportion of DEGs could not be annotated by COG categories, more functional genes were apparently transcribed differentially in #22 than that of #40 across the sampling duration (Figure 3E), indicating that the mutation H527R made more significant contributions on the alterations of global gene transcriptional profile in Ni1-3.

### Gene Transcriptional Profiles Correlated to Extracellular Respiration

**FIGURE 4:**
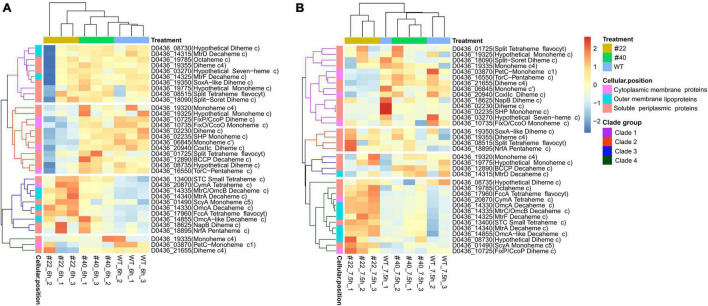
Comparison of the expression level of cytochrome *c* coding genes between the strains. Expression level of cytochrome *c* coding genes at 6 h **(A)** and 7.5 h **(B)**. Sample #22_6h_2 is an outlier **(A)**.

It has long been appreciated that numerous exoelectrogens contain multi-heme cytochromes, which enable the bacteria to respirate various substrates, thus being competent for bioremediation (Breuer et al., 2015). To date, approximately 42 multi-heme cytochrome *c* proteins have been identified in *S. oneidensis* MR-1 (Meyer et al., 2004; Fang et al., 2019). Among them, the CymA-Mtr EET pathway has been proven to play vital roles in extracellular reduction, of which CymA, OmcA, MtrC, MtrF, etc. are the key cytochromes, and the substrates could be graphite anodes, ferric citrate, hydroxides of Fe (III) or Mn (IV), azo dyes, and so on (Clarke et al., 2016; Zhang et al., 2020). In total, 35 cytochrome *c* coding sequences were identified in the genome of *S. decolorationis* Ni1-3 by homological sequence alignment (coverage > 90%, identity > 80%, and *e*-value < 1 × e^–5^). Overall, the transcriptional profiles of 35 cytochrome *c* genes in #22 were much different from that in #40 and Ni1-3, which could divide the samples into two major clades (Figure 4). Apparently, the cytochrome *c* genes could be clustered into four clades at both 6 and 7.5 h in the strains (Figure 4), the cytochrome *c* genes in clades 2 and 4 at 6 h and in clade 1 at 7.5 h encode soluble periplasmic and cytoplasmic membrane proteins, which showed much lower transcription levels in #22 than in #40 and Ni1-3 (Figure 4). The result suggested that RNAP mutation in #22 may result in the defect of bio-redox reactions occurring in the periplasm of Ni1-3, and the bioremediation activity of pollutants which could pass the outer membrane will be affected. More importantly, as these genes were downregulated in #22, which was identical to the AMR degradation capacity between strains, they might be associated with azo dye degradation. Conversely, the cytochrome *c* genes in clade 3 at 6 h and in clade 4 at 7.5 h, coding for CymA-Mtr and other functionally important cytochrome *c* proteins, exhibited higher transcriptional levels in #22 when compared to #40 and Ni1-3 (Figure 4). For instance, genes coding for CymA, OmcA, MtrC, and MtrA were upregulated in #22, which would enhance the azo dye biodegradation and oxidized-metal reduction according to the identified functions of the classic CymA-Mtr EET pathway. Unexpectedly, although the enhanced leaching capacity was observed in #22, the AMR biodegradation was severely defected (Figure 2), implying that *S. decolorationis* Ni1-3 could degrade AMR mostly via an exclusive way.

### Gene Co-expression Network Analysis for AMR Degradation

**FIGURE 5:**
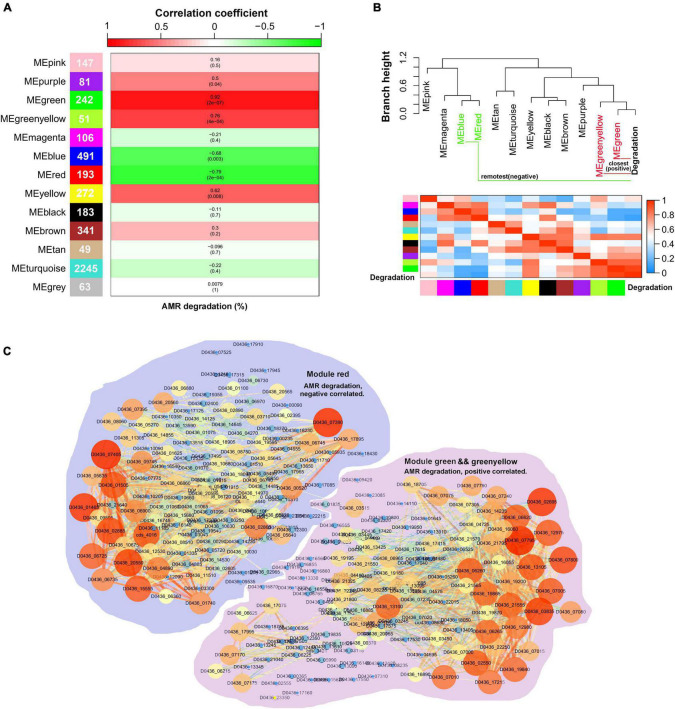
Visualization of the correlations between the co-expression gene modules and the sample traits. Numbers in the cells of the left color bar represent gene numbers contained in the corresponding modules. The top color bar represents the module–trait correlation coefficient ranging from -1 to 1. The color in the cells of the middle columns represents the correlation marked by the top color bar, numbers in the brackets were the *p*-value, and numbers outside the brackets were the correlation coefficient. A combination of *p* ≤ 0.05 and correlation coefficient ≥ 0.75 or ≤ -0.75 were identified as significantly correlated pairs **(A)**. The module–trait dendrogram and adjacency heatmap **(B)**. Co-expression networks of the AMR degradation correlated gene modules. Node size in the network represents its connectivity; the bigger the node size, the more the connections. Node label color represents the genes’ module membership (MM), the higher MM is displayed by the brighter color **(C)**.

As *S. decolorationis* Ni1-3 may degrade AMR in an exclusive way, WGCNA was conducted to explore gene networks correlated to AMR biodegradation. Sample #22_6h_2 was removed as an outlier, and the soft power for calculating the scale-free network was determined to be seven to meet the rules of scale-free network construction (Supplementary Figure 5). The whole transcriptome of Ni1-3 was finally clustered into 13 co-expressed gene modules (Supplementary Figure 6), of which the smallest module contained 49 genes and the largest module contained 2,245 genes (Figure 5A). By conducting a module–trait correlation analysis, we found that modules green and green-yellow were positively correlated to AMR degradation while module red was negatively correlated to AMR degradation (Figure 5A). As shown in Figure 5B, the modules and AMR degradation could be divided into two major clades (cut-height = 1.0), the closest two modules to AMR degradation were modules green and green-yellow, while the remotest were modules blue and red (Figure 5B). Interestingly, 11 out of the 35 cytochrome *c* proteins mainly in the CymA-Mtr pathway were clustered into module blue, which showed a weak correlation to AMR biodegradation. Only one cytochrome *c* protein (TorC) located in the cytoplasmic membrane was clustered into module green and showed a high GS value of 0.85 to AMR degradation (Supplementary Table 2), suggesting that this cytochrome *c* protein might be a particularly important component in the anaerobic respiration chain of AMR degradation as AMR could not penetrate the cell with the highly polarized sulfate groups. Besides, genes in module blue code for proteins involved in various substrate utilizations and are important to bioremediation as the main CymA-Mtr pathway-correlated cytochrome *c* coding genes were clustered/co-expressed in this module (Supplementary Table 2).

Because they were significantly correlated to AMR degradation, the networks of modules red, green, and green-yellow were visualized using Cytoscape (Figure 5C). By extracting both important genes in the networks and co-expression modules, a hub gene set negatively or positively correlated to AMR degradation was obtained. The total number of hub genes in the set was 71, of which 35 belonged to module green, 6 belonged to module green-yellow, and 30 belonged to module red (Supplementary Table 3). However, only 18 hub genes were differentially expressed at the transcriptional level. Nine of the DEGs belonged to module green; one belonged to module green-yellow, which was positively correlated to AMR degradation; and eight belonged to module red, which were negatively correlated to AMR degradation (Table 1). Notably, some of the differentially expressed hub genes had been proven to be associated with anaerobic bioreduction in *Shewanella* spp. For instance, YceI and YceJ were downregulated in the negative mutant #22, which has been reported to be involved in the extracellular anaerobic bioreduction of 2,5-dichloronitrobenzene and acted as part of the components to transfer electron to the non-specific outer-membrane reductase (Clarke et al., 2016; Cao et al., 2017; Zhang et al., 2020). Sye4, downregulated in #22 at 7.5 h, is a member of the Old Yellow Enzyme family, which has been identified as a versatile FMN-dependent NADPH oxidoreductase (Elegheert et al., 2010). TorC, downregulated in #22 at 7.5 h, has been identified as an inner-membrane cytochrome *c* protein involved in TMAO reduction (Breuer et al., 2015). Besides, extradiol ring-cleavage dioxygenase, encoded by D0436_09400, was downregulated in #22 at 7.5 h and may be involved in the biodegradation of aromatic compounds relevant to azo dye degradation (Eslami et al., 2016). However, some other differentially expressed hub genes had no proofs involved in the extracellular anaerobic bioreduction (Table 1). For instance, CydA, upregulated in #22 at 6 h, has been proven to play important roles in stress adaptation (e.g., nitrite resistance) under aerobic growth (Chen et al., 2015). PhrB, downregulated in #22 at 6 h, was a photolyase-related protein (Table 1). These results suggested that AMR degradation in Ni1-3 may substantially depend on non-specific outer-membrane reductase, and mutations in the RNAP may alter gene transcriptional profiles in the AMR degradation process and the relevant aromatic compound degradation process.

**TABLE 1:** Characterization of the differentially expressed hub genes.

Hub_genes	Modules	GS degradation	6 h (#22 vs. WT) logFC	6 h (#40 vs. WT) logFC	7.5 h (#22 vs. WT) logFC	7.5 h (#40 vs. WT) logFC	Gene symbol	Function
D0436_07080	Green	0.93	−1.02	−0.01	−1.60	−0.11	*yceI*	YceI family protein
D0436_09400	Green	0.92	−0.64	−0.03	−1.86	−0.13	NA	Extradiol ring-cleavage dioxygenase, class III enzyme, subunit B
D0436_07075	Green	0.92	−0.85	0.07	−1.45	0.22	*yceJ*	PFAM cytochrome *b*_561_
D0436_06965	Green	0.89	−0.56	−0.32	−1.43	−0.10	*sye4*	PFAM NADPH flavin oxidoreductase
D0436_19200	Green	0.88	−1.04	−0.09	−2.16	0.63	NA	Belongs to the resistance- nodulation-cell division (RND) (TC 2.A.6) family
D0436_16550	Green	0.85	−0.64	−0.28	−1.10	0.23	*torC*	Belongs to the TorC TorY family
D0436_07005	Green	0.83	−1.19	−0.08	−1.02	0.90	NA	SnoaL-like domain
D0436_19865	Green	0.82	−1.11	0.04	−0.86	0.89	*phrB*	Deoxyribodipyrimidine photolyase-related protein
D0436_07010	Green	0.75	−1.15	−0.08	−1.05	0.85	NA	Short-chain dehydrogenase reductase SDR
D0436_16345	Green-yellow	0.66	0.75	0.95	0.51	1.37	*dnaG*	RNA polymerase that catalyzes the synthesis of short RNA molecules used as primers for DNA polymerase during DNA replication
D0436_06725	Red	−0.66	1.07	0.69	0.72	0.37	*ompK*	PFAM nucleoside-specific channel-forming protein, Tsx
D0436_02885	Red	−0.76	2.16	1.22	1.58	0.14	NA	NA
D0436_20550	Red	−0.78	1.75	0.96	1.26	0.20	NA	TIGRFAM formate dehydrogenase region TAT target
D0436_07390	Red	−0.80	1.01	0.47	0.92	0.48	*cydA*	PFAM cytochrome *bd* ubiquinol oxidase subunit I
D0436_07405	Red	−0.82	1.06	0.39	0.92	0.26	NA	Histidine kinase, HAMP region domain protein
D0436_15555	Red	−0.83	1.35	0.64	1.18	0.30	NA	NA
D0436_05820	Red	−0.84	1.10	0.51	1.18	0.20	NA	Prokaryotic cytochrome *b*_561_
D0436_05640	Red	−0.94	0.97	0.20	1.07	−0.09	*cmpR*	Transcriptional regulator, LysR family

*GS degradation indicates the gene significance of the module gene to the azo dye degradation efficiency, which was calculated using the WGCNA software and represented the correlation coefficient between the module gene and the azo dye degradation efficiency. FC indicates the fold change of the specific gene between the mutants and the WT at the transcriptional level at different sampling time points.*

### Cellular Model of Azo Dye Degradation

**FIGURE 6:**
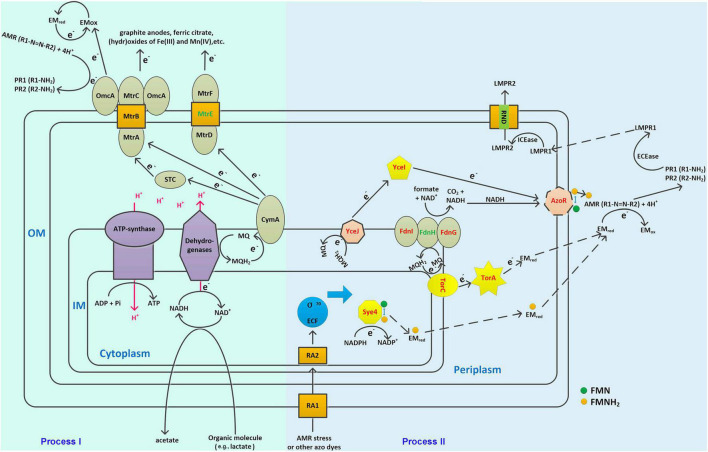
Schematic cellular model of the novel identified azo dye degradation pathway in *S. decolorationis*. OM, IM, MQ, EMred, EMox, ECEase, ICEase, LMPR1, LMP2, RA1, and RA2 represent outer membrane, inner membrane, menaquinone, electron mediator in the reduced state, electron mediator in the oxidized state, extracellular enzymes, intracellular enzymes, low-molecular product-1, low-molecular product-2, stress response protein-1, and stress response protein-2, respectively. Proteins colored in red are key components in the hypothesized azo dye biodegradation models. Proteins colored in green are those not identified in the Ni1-3 genome. The solid arrows represent reactions or electron transfer pathways that have been determined previously. The dotted arrows represent reactions or electron transfer pathways hypothesized in this study.

Apart from the hub genes, some other genes in modules green, red, and green-yellow may also be involved in AMR degradation, which could accept electrons from the hub gene coded proteins. For instance, AzoR (D0436_01835, module green) showed a GS of 0.91 to AMR degradation, which has been identified as non-specific FMN-dependent NADH azo reductase and could accept electron from the YceJ–YceI chain to degrade extracellular 2,5-dichloronitrobenzene (Zhang et al., 2020). Notably, YceI and YceJ showed GS values higher than 0.9 to AMR degradation, suggesting that this EET pathway played vital roles in AMR degradation. In addition, TorA (D0436_16555, module green) showed a GS of 0.82 to AMR degradation, which has been identified as a TMAO reductase in bacteria and can accept electron from the inner-membrane-bounded TorC (Mintmier et al., 2020). The protein–protein interactions explored by using STRING further confirmed the strong interactions between these proteins (Supplementary Figure 7). As shown in Supplementary Figure 7, YceI interacts with YceJ (combined score 0.970, high confidence) (Supplementary Figure 7A), AzoR interacts with Sye4 (combined score 0.712, high confidence) (Supplementary Figure 7B), and TorA interacts with TorC, TorD, and the formate dehydrogenase subunits (e.g., FdnI, FdnG, and FdnH) (combined score higher than 0.910, high confidence) (Supplementary Figure 7C). A previous study by Zhang et al. (2020) has also observed the co-expression between *azoR* and *sye4*, although the mechanisms have not been determined. It has been determined that AzoR and Sye4 are both flavin enzymes (FMN non-covalent bond) which are NADH and NADPH dependent, respectively^6^; these two proteins may act as isozymes to degrade azo dyes. However, Sye4 is a periplasm protein; it could only degrade AMR indirectly. As FMN non-covalently bound to Sye4, the reduced FMNH_2_ may be released from the binding site and act as an electron mediator to cross the membranes (Figure 6). Moreover, the interactions between TorC/TorA and the formate dehydrogenases have been determined in *E. coli* K12 in the formate to trimethylamine N-oxide electron transfer pathway^7^. Meanwhile, the GS values of *fdnI* and *fdnG* were both higher than 0.63 (*p* ≤ 0.01) (data not shown), indicating a significantly positive correlation to AMR degradation. Consequently, experiments were conducted to verify the involvement of TorA in azo dye degradation (see Supplementary Materials and Methods section “Verification of the TorA Involvement in Azo Dye Degradation”). Results showed that TorA was positively correlated to azo dye degradation (Supplementary Figure 8). Besides, some sigma factors have been found to be positively correlated with AMR biodegradation. For instance, four sigma-factor-related genes in module green were observed to be significantly correlated with AMR degradation. Among them, two (i.e., D0436_05260 GS 0.78 and D0436_13105 GS 0.85) were anti-sigma factors positively correlated with AMR degradation. D0436_13105 encodes ChrR that has been identified as a σ*^24^* anti-factor (Mauro et al., 2019; Lemke et al., 2020). One (i.e., D0436_03030 GS -0.92) was σ*^D^* (i.e., σ^70^) a regulator that negatively correlated with AMR degradation. D0436_03030 encodes RSD that acts as an anti-σ factor antagonist (Bouillet et al., 2018), which means that the high expression of D0436_03030 can bind more anti-σ factors, thus promoting the function of σ^70^. One was σ*^K^* (i.e., D0436_13100 GS 0.78) that positively correlated with AMR degradation. D0436_13100 belongs to the σ^70^ ECF subfamily, which responded to various environmental stress (Fang et al., 2019). Consequently, *S. decolorationis* Ni1-3 may enhance the transcriptional levels of AMR degradation-related genes through responses to AMR stress, and these genes are mainly transcribed by σ^70^ ECF subfamily factor-bounded promotors. Consistently, some stress-associated genes such as molecular chaperone DnaK/J and several efflux RND transporters were found downregulated in #22, which had been reported as associated with stress responses and detoxification by expelling the toxic degradation products into extracellular in *S. decolorationis* S12 (Fang et al., 2019). Thus, multiple pathways could be concluded to biodegrade azo dyes, one was the classic CymA-Mtr pathway (Process I), and the others were non-CymA-Mtr pathways (Process II). As we have observed in this study, Process I was downregulated whereas Process II was upregulated in WT and #40, those with higher AMR degradation capacity when compared to that of #22, indicating that Process II dominates azo dye degradation in *S. decolorationis* Ni1-3. Process II starts from the response of azo dye stress; then, genes initiating with σ^70^ ECF subfamily promotors were stimulated to transcribe at high levels. Meanwhile, electron from the cellular menaquinone (MQ) pool was sequentially transferred to the inner-membrane cytochrome *c* proteins (e.g., TorC) or cytochrome *b* proteins (e.g., YceJ), periplasm electron mediator enzymes (e.g., TorA or YceI) or electron mediator chemicals, electron mediator chemicals or non-specific outer-membrane azo reductase (e.g., AzoR), or extracellular electron mediators to start azo dye degradation. In the process, oxidoreductase, especially flavin-dependent oxidoreductase on the downstream of the electron transfer chains, played vital roles. After that, the aromatic compounds produced from azo dye degradation could be further degraded by various extracellular or intracellular enzymes, and the intracellular toxic products could be exported to extracellular through efflux RND transporters (Figure 6).

## Conclusion

A total of 11 RNAP mutants of *S. decolorationis* Ni1-3 were screened out in this study, of which some showed promising potential in the bioremediation of azo dye or e-waste (e.g., spent LIBs) pollution. Based on the RNA-seq analysis, global transcriptional profile alternations were observed resulting from the RNAP mutation. Genes in Ni1-3 could be clustered into 13 co-expression gene modules, of which two were positively correlated while one was negatively correlated with AMR degradation. Moreover, non-CymA-Mtr-dominated AMR degradation EET pathways (i.e., TorC-TorA- or YceJ-YceI-based EET pathways) were identified in *S. decolorationis* Ni1-3, which started from the azo dye stress response proteins (SRP) in the outer membrane and coupled with intracellular stress response, thus regulating the σ^70^ ECF subfamily factors to stimulate downstream gene transcriptional profiles. Then, electron transfer started from the MQ pool to the inner-membrane cytochrome *c/b* proteins and completed by electron mediator chemicals or non-specific outer-membrane azo reductase. The results prove that RNAP mutation is feasible to obtain *S. decolorationis* mutants with enhanced bioremediation potential. Especially the application of *S. decolorationis* strains in the bioleaching of spent LIBs would greatly shorten the leaching cycles (70–90%) and save the costs (low-nutrient medium and no requirement of inorganic acids) compared to traditional bioleaching methods. Besides, the conceptualized cellular model gives novel insights toward azo dye degradation pathways as well as the gene co-expression networks.

## Data Availability Statement

The datasets presented in this study can be found in online repositories. The names of the repository/repositories and accession number(s) can be found below: https://www.ncbi.nlm.nih.gov/, PRJNA698259 and https://www.ncbi.nlm.nih.gov/genbank/, CP031775.

## Author Contributions

XC and XZ performed the mutants isolation, RNA-seq, and manuscript preparation. YW and LT performed the RNA-seq based analysis, and reviewed and revised the manuscript. YM designed the whole study. All authors made substantial and direct contributions to the work, and read and approved the final manuscript.

## Conflict of Interest

The authors declare that the research was conducted in the absence of any commercial or financial relationships that could be construed as a potential conflict of interest.

## Publisher’s Note

All claims expressed in this article are solely those of the authors and do not necessarily represent those of their affiliated organizations, or those of the publisher, the editors and the reviewers. Any product that may be evaluated in this article, or claim that may be made by its manufacturer, is not guaranteed or endorsed by the publisher.

## Footnotes

1
https://github.com/FelixKrueger/TrimGalore

2
https://github.com/samtools/samtools

3
https://github.com/simon-anders/htseq

4
https://github.com/eggnogdb/eggnog-mapper

5
http://string-db.org/

6
https://www.uniprot.org/uniprot/

7
https://biocyc.org/ECOLI/NEW-IMAGE?type=PATHWAY&object=PWY0-1355&REG-DETAIL=T
