# Supplementary material for: Enhanced Bioremediation Potential of Shewanella decolorationis RNA Polymerase Mutants and Evidence for Novel Azo Dye Biodegradation Pathways
Source: Front Microbiol. 2022 Mar 22;13:843807. doi: 10.3389/fmicb.2022.843807 (PMC8981235; doi:10.3389/fmicb.2022.843807)
Supplement: Supplementary file 1 [file Data_Sheet_1.pdf]

Supplementary information for

Enhanced bioremediation potential of *Shewanella decolorationis* RNA polymerase mutants and evidence for novel azo dye biodegradation pathways

Xunchao Cai[1,2,†], Xin Zheng[1,†], Yicheng Wang[1], Li Tian[1], Yanping Mao[1,*]

1. College of Chemistry and Environmental Engineering, Shenzhen University, Shenzhen, Guangdong, 518071, China
2. Department of Gastroenterology and Hepatology, Shenzhen University General Hospital, Shenzhen, Guangdong, 518071, China

[†]Xunchao Cai and Xin Zheng contributed equally to this work.

*Corresponding author: Tel: +86-755-26558094; E-mail address: maoy@szu.edu.cn (Y. Mao)

Submitted to Frontiers in Microbiology

The supplementary information contains 3 tables, 8 figures and supplementary materials and methods.

Table Captions and Figure Legends

Table S1. Characterization of point mutations in the *rpoB* gene
Table S2. Module traits of the cytochrome-c genes
Table S3. Hub genes in the modules significantly correlated to AMR degradation

Figure S1. The chemical structural formula of amaranth (AMR). This compound is highly polarized by three sulfonate groups.
Figure S2. Growth curves of *S. decolorationis* Ni1-3 and the RNAP mutants in LB medium.
Figure S3. Aerobic degradation of AMR by *S. decolorationis* Ni1-3 and RNAP mutants. The initial concentration of AMR was 8 mM. Incubation conducted aerobically on 37 °C after 460 min (A), 540 min (B), 600 min (C) and 780 min (D).
Figure S4. Pairwise scatter (A) and multidimensional scaling (MDS) (B) plot of the counts data.
Figure S5. Soft-power pick for the construction of scale free network model.
Figure S6. Merged dynamic tree of the gene module.
Figure S7. Analysis of protein-protein interaction networks using STRING. Network line thickness indicates the strength of data support. Combined score higher than 0.700 are shown in the figure. Network nodes marked with brown shadow are formate dehydrogenase subunits.
Figure S8. Phenotype verification of the key protein involvement in the suggested azo dye degradation pathway in *S. decolorationis*. (A). Inhibition of AMR degradation by adding sodium tungstate. (B). Comparison of the TMAO reduction efficiencies between the WT strain and the mutants.

Supplementary tables

Table S1. Characterization of point mutations in the *rpoB* gene

Mutants	Mutations in *rpoB*	Mutations in β subunit (RpoB)	Proportion (%)	Number of clones
#6	G692T	V146F	19.15%	9
#25	A1541T	Q514L	4.26%	2
#46	A1541G	Q514R	2.13%	1
#40	A1550T	D517V	6.38%	3
#18	C1568T	S523L	10.64%	5
#28	C1579G	H527D	6.38%	3
#36	C1579T	H527Y	12.77%	6
#22	A1580G	H527R	4.25%	2
#21	G1589A	R530H	8.51%	4
#13	C1595T	S532F	23.40%	11
#26	C1595A	S532Y	2.13%	1

删除标识 | 福昕编辑器

福昕PDF编辑器 福昕PDF编辑器 福昕PDF编辑器

42

Table S2. Module traits of the cytochrome-c genes

GeneID	name	moduleColor	GS.Decolorization	p.GS.Decolorization	Function
D0436_06845	cytochrome *c*	blue	0.420265	0.093027979	Monoheme *c*
D0436_08515	cytochrome *c*	blue	-0.41745	0.095470418	Split Tetraheme flavocyt
D0436_14315	DmsE family decaheme *c*-type cytochrome	blue	0.487092	0.047361463	MtrD Decaheme *c*
D0436_14325	OmcA/MtrC family decaheme *c*-type cytochrome	blue	-0.46839	0.057917717	MtrF Decaheme *c*
D0436_14330	OmcA/MtrC family decaheme *c*-type cytochrome	blue	-0.6665	0.003481306	OmcA Decaheme *c*
D0436_14335	OmcA/MtrC family decaheme *c*-type cytochrome	blue	-0.59149	0.012387616	MtrC/OmcB Decaheme *c*
D0436_14340	DmsE family decaheme *c*-type cytochrome	blue	-0.57038	0.016809788	MtrA Decaheme *c*
D0436_17960	flavocytochrome *c*	blue	-0.77775	0.000237502	FccA Tetraheme flavocyt
D0436_18625	nitrate reductase cytochrome *c*-type subunit	blue	-0.53178	0.0280177	NapB Diheme *c*
D0436_18895	ammonia-forming nitrite reductase cytochrome c_{552} subunit	blue	-0.56396	0.018376286	NrfA Pentaheme *c*
D0436_19785	tetrathionate reductase family octaheme c-type cytochrome	blue	-0.57803	0.015083796	Octaheme *c*
D0436_20870	cytochrome *c*	blue	-0.63108	0.006594976	CymA Tetraheme *c*
D0436_01490	cytochrome c_5 family protein	brown	-0.46224	0.061745784	ScyA Monoheme c_5
D0436_12890	cytochrome *c* peroxidase	brown	0.280056	0.276278131	BCCP Decaheme *c*
D0436_21655	cytochrome c_4	brown	0.265841	0.302394899	Diheme c_4
D0436_16550	pentaheme *c*-type cytochrome TorC	green	0.850361	1.53E-05	TorC-Pentaheme *c*
D0436_19325	cytochrome *c*	grey	-0.01611	0.951060422	Hypothetical Monoheme *c*
D0436_19335	cytochrome *c*	grey	0.012186	0.962978267	Monoheme c_4
D0436_19775	cytochrome *c*	pink	0.09226	0.72470574	Hypothetical Monoheme *c*

删除标识 | 福昕编辑器
福昕PDF编辑器

D0436_02230	diacylglycerol kinase	purple	0.411696	0.100602199	Diheme *c*
D0436_02235	DUF1924 domain-containing protein	purple	0.396524	0.115076011	SHP Monoheme *c*
D0436_14855	OmcA/MtrC family decaheme *c*-type cytochrome	red	-0.72454	0.001002706	OmcA-like Decaheme *c*
D0436_18090	cytochrome *c*	red	-0.61071	0.009213097	Split-Soret Diheme *c*
D0436_19355	cystathionine beta-synthase	red	-0.62266	0.007593365	Diheme c_4
D0436_03270	cytochrome *c*	turquoise	-0.45701	0.065144908	Hypothetical Seven-heme *c*
D0436_03870	cytochrome c_1	turquoise	0.097922	0.708486965	PetC-Monoheme c_1
D0436_08730	hypothetical protein	turquoise	-0.2053	0.429242318	Hypothetical Diheme *c*
D0436_08735	hypothetical protein	turquoise	-0.07611	0.771560601	Hypothetical Diheme *c*
D0436_10725	cytochrome-*c* oxidase, cbb_3-type subunit III	turquoise	-0.03813	0.884471979	FixP/CcoP Diheme *c*
D0436_10735	cytochrome-*c* oxidase, cbb_3-type subunit II	turquoise	0.007738	0.976486251	FixO/CcoO Monoheme *c*
D0436_13400	cytochrome *c*	turquoise	-0.20859	0.42173296	STC Small Tetraheme *c*
D0436_19320	cytochrome *c*	turquoise	-0.18793	0.470106091	Monoheme c_4
D0436_19350	cytochrome *c*	turquoise	-0.63988	0.005667275	SoxA-like Diheme *c*
D0436_20940	cytochrome c oxidase subunit II	turquoise	-0.01877	0.942993178	CoxIIc Diheme *c*
D0436_01725	cytochrome *c*	yellow	0.273021	0.289025954	Split Tetraheme flavocyt

Table S3. Hub genes in the modules significantly correlated to AMR degradation

Hub_genes	Method	Modules	GS.Decolorization	p.GS.Decolorization	Symbol	Functions
D0436_02550	both	green	0.876536	3.92E-06	*NA*	NA
D0436_02695	both	green	0.951397	4.5E-09	*yihG*	PFAM phospholipid glycerol acyltransferase
D0436_03835	both	green	0.86661	6.79E-06	*miaA*	Catalyzes the transfer of a dimethylallyl group onto the adenine at position 37 in

D0436_06260	network connectivity	green	0.781597	0.000211	*dnaK*	Heat shock 70 kDa protein
D0436_06265	MM && GS	green	0.828547	3.97E-05	*dnaJ*	ATP binding to DnaK triggers the release of the substrate protein, thus completing
D0436_06620	network connectivity	green	0.788371	0.00017	*ftsJ*	Specifically methylates the uridine in position 2552 of 23S rRNA at the 2'-O posit
D0436_06765	MM && GS	green	0.857076	1.11E-05	*recX*	Modulates RecA activity
D0436_06960	MM && GS	green	0.903981	6.46E-07	*NA*	PFAM regulatory protein TetR
D0436_06965	MM && GS	green	0.885761	2.25E-06	*sye4*	PFAM NADH flavin oxidoreductase NADH oxidase
D0436_07005	both	green	0.832011	3.45E-05	*NA*	SnoaL-like domain
D0436_07010	network connectivity	green	0.751243	0.000508	*NA*	PFAM short-chain dehydrogenase reductase SDR
D0436_07075	MM && GS	green	0.920392	1.66E-07	*yceJ*	PFAM cytochrome B561
D0436_07080	MM && GS	green	0.934475	4.02E-08	*yceI*	PFAM YceI family protein
D0436_07790	MM && GS	green	0.956819	1.88E-09	*NA*	NA
D0436_07795	network connectivity	green	0.934594	3.97E-08	*vacJ*	PFAM VacJ family lipoprotein
D0436_07800	both	green	0.95036	5.26E-09	*NA*	PFAM response regulator receiver
D0436_09400	MM && GS	green	0.920861	1.59E-07	*NA*	PFAM Extradiol ring-cleavage dioxygenase, class III enzyme, subunit B
D0436_12975	both	green	0.828327	4.01E-05	*ybbN*	PFAM Thioredoxin domain
D0436_13100	network connectivity	green	0.783934	0.000196	*sigK*	RNA polymerase, sigma-24 subunit, ECF subfamily
D0436_13105	both	green	0.847404	1.76E-05	*chrR*	TIGRFAM anti-sigma factor
D0436_13425	MM && GS	green	0.939548	2.23E-08	*NA*	Protein of unknown function (DUF3187)
D0436_14235	MM && GS	green	0.820456	5.48E-05	*lon*	ATP-dependent serine protease that mediates the selective degradation of mutant an
D0436_16060	MM && GS	green	0.854605	1.25E-05	*rseB*	PFAM MucB RseB family protein
D0436_16550	MM && GS	green	0.850361	1.53E-05	*torC*	Belongs to the TorC TorY family

D0436_17215	MM && GS	green	0.800034	0.000115	*clpB*	Part of a stress-induced multi-chaperone system, it is involved in the recovery of
D0436_17415	MM && GS	green	0.91909	1.87E-07	*NA*	PFAM aminoglycoside phosphotransferase
D0436_19200	MM && GS	green	0.884487	2.44E-06	*NA*	Belongs to the resistance-nodulation-cell division (RND) (TC 2.A.6) family
D0436_19840	network connectivity	green	0.768566	0.000313	*hslU*	this subunit has chaperone activity. The binding of ATP and its subsequent hydroly
D0436_19865	both	green	0.824539	4.67E-05	*phrB*	PFAM deoxyribodipyrimidine photolyase-related protein
D0436_21325	MM && GS	green	0.867464	6.49E-06	*acrB*	TIGRFAM transporter, hydrophobe amphiphile efflux-1 (HAE1) family
D0436_21550	MM && GS	green	0.85223	1.4E-05	*arnT*	Dolichyl-phosphate-mannose-protein mannosyltransferase
D0436_21555	both	green	0.887693	1.99E-06	*NA*	SNARE associated Golgi protein
D0436_21560	MM && GS	green	0.875039	4.27E-06	*arnC*	Glycosyltransferase like family 2
D0436_21565	MM && GS	green	0.938123	2.65E-08	*ugd*	Belongs to the UDP-glucose GDP-mannose dehydrogenase family
D0436_21570	MM && GS	green	0.93649	3.2E-08	*capI*	PFAM NAD-dependent epimerase dehydratase
D0436_11020	network connectivity	greenyell ow	0.687738	0.002279	*rnfB*	Part of a membrane complex involved in electron transport
D0436_11660	MM && GS	greenyell ow	0.804653	9.82E-05	*rluE*	Belongs to the pseudouridine synthase RsuA family
D0436_14245	MM && GS	greenyell ow	-0.84599	1.87E-05	*clpP*	Cleaves peptides in various proteins in a process that requires ATP hydrolysis. Ha
D0436_16345	network connectivity	greenyell ow	0.657105	0.004156	*dnaG*	RNA polymerase that catalyzes the synthesis of short RNA molecules used as primers
D0436_17795	MM && GS	greenyell ow	0.81853	5.9E-05	*fecD*	Belongs to the binding-protein-dependent transport system permease family. FecCD s

D0436_19705	network connectivity	greenyellow	0.792642	0.000148	*ftsN*	PFAM Sporulation domain protein
D0436_00520	MM && GS	red	-0.93541	3.62E-08	*Z012_04450*	Belongs to the pirin family
D0436_01465	MM && GS	red	-0.708	0.001472	*ccmE*	Heme chaperone required for the biogenesis of c-type cytochromes. Transiently bind
D0436_01505	MM && GS	red	-0.80051	0.000113	*dsbE*	TIGRFAM periplasmic protein thiol--disulphide oxidoreductase DsbE
D0436_02865	MM && GS	red	-0.87504	4.27E-06	*frdB*	TIGRFAM succinate dehydrogenase and fumarate reductase iron-sulfur protein
D0436_02885	MM && GS	red	-0.76406	0.000356	*NA*	
D0436_03515	MM && GS	red	-0.95308	3.47E-09	*NA*	PFAM beta-lactamase domain protein
D0436_04655	MM && GS	red	-0.84866	1.66E-05	*NA*	RmlD substrate binding domain
D0436_04890	both	red	0.808533	8.55E-05	*purA*	Plays an important role in the de novo pathway of purine nucleotide biosynthesis.
D0436_05330	MM && GS	red	-0.89924	9.14E-07	*kdsA*	3-deoxy-D-manno-octulosonic acid 8-phosphate synthase
D0436_05640	MM && GS	red	-0.9381	2.65E-08	*cmpR*	Transcriptional regulator, LysR family
D0436_05820	MM && GS	red	-0.8354	2.99E-05		Prokaryotic cytochrome b561
D0436_06725	MM && GS	red	-0.66444	0.003621	*ompK*	PFAM nucleoside-specific channel-forming protein, Tsx
D0436_06735	MM && GS	red	-0.78187	0.000209	*deoA*	The enzymes which catalyze the reversible phosphorolysis of pyrimidine nucleosides
D0436_06740	MM && GS	red	-0.84203	2.24E-05	*deoB*	Phosphotransfer between the C1 and C5 carbon atoms of pentose
D0436_06745	MM && GS	red	-0.90962	4.17E-07	*deoD*	TIGRFAM purine nucleoside phosphorylase
D0436_07390	MM && GS	red	-0.80178	0.000109	*cydA*	PFAM cytochrome bd ubiquinol oxidase subunit I

D0436_07395	MM && GS	red	-0.84929	1.61E-05	*cydB*	TIGRFAM cytochrome d ubiquinol oxidase, subunit II
D0436_07405	MM && GS	red	-0.82269	5.02E-05	*NA*	PFAM histidine kinase, HAMP region domain protein
D0436_08750	MM && GS	red	-0.81389	7.03E-05	*NA*	Protein of unknown function (DUF3083)
D0436_09850	MM && GS	red	-0.81611	6.47E-05	*NA*	TonB-dependent receptor
D0436_10310	MM && GS	red	-0.82522	4.54E-05	*ssb*	Plays an important role in DNA replication, recombination and repair. Binds to ssD
D0436_10540	MM && GS	red	0.801006	0.000112	*lrp*	Regulatory protein AsnC Lrp family
D0436_12300	MM && GS	red	-0.84372	2.08E-05	*doc*	Death ON curing protein
D0436_13650	MM && GS	red	-0.91781	2.1E-07	*NA*	PFAM short-chain dehydrogenase reductase SDR
D0436_14230	MM && GS	red	-0.84939	1.6E-05	*hupB*	Histone-like DNA-binding protein which is capable of wrapping DNA to stabilize it,
D0436_15555	MM && GS	red	-0.82879	3.93E-05	*NA*	NA
D0436_17965	MM && GS	red	-0.88202	2.83E-06	*ldhA*	PFAM D-isomer specific 2-hydroxyacid dehydrogenase, catalytic region
D0436_19595	MM && GS	red	-0.82186	5.19E-05	*NA*	Prokaryotic N-terminal methylation motif
D0436_20550	MM && GS	red	-0.77739	0.00024	*NA*	TIGRFAM formate dehydrogenase region TAT target
D0436_21640	MM && GS	red	-0.88586	2.24E-06	*elbB*	Displays glyoxalase activity, catalyzing the conversion of glyoxal to glycolate

Supplementary figures

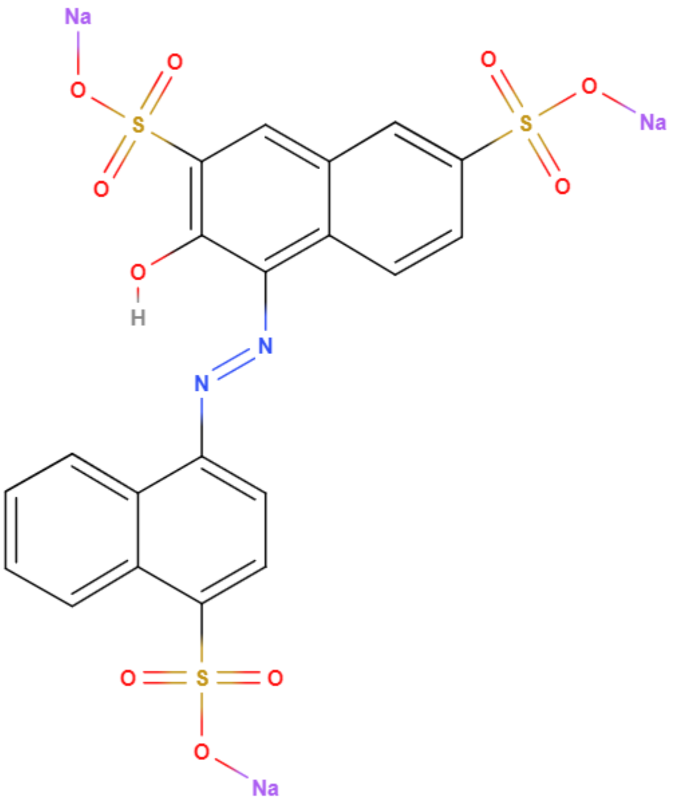

Figure S1. The chemical structural formula of amaranth (AMR). This compound is highly polarized by three sulfonate groups.

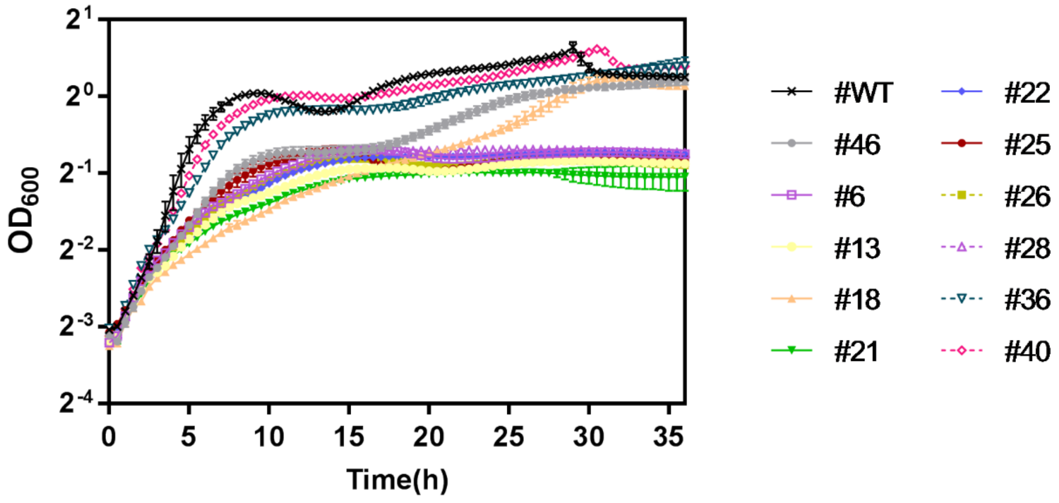

Figure S2. Growth curves of *S. decolorationis* Ni1-3 and the RNAP mutants in LB medium.

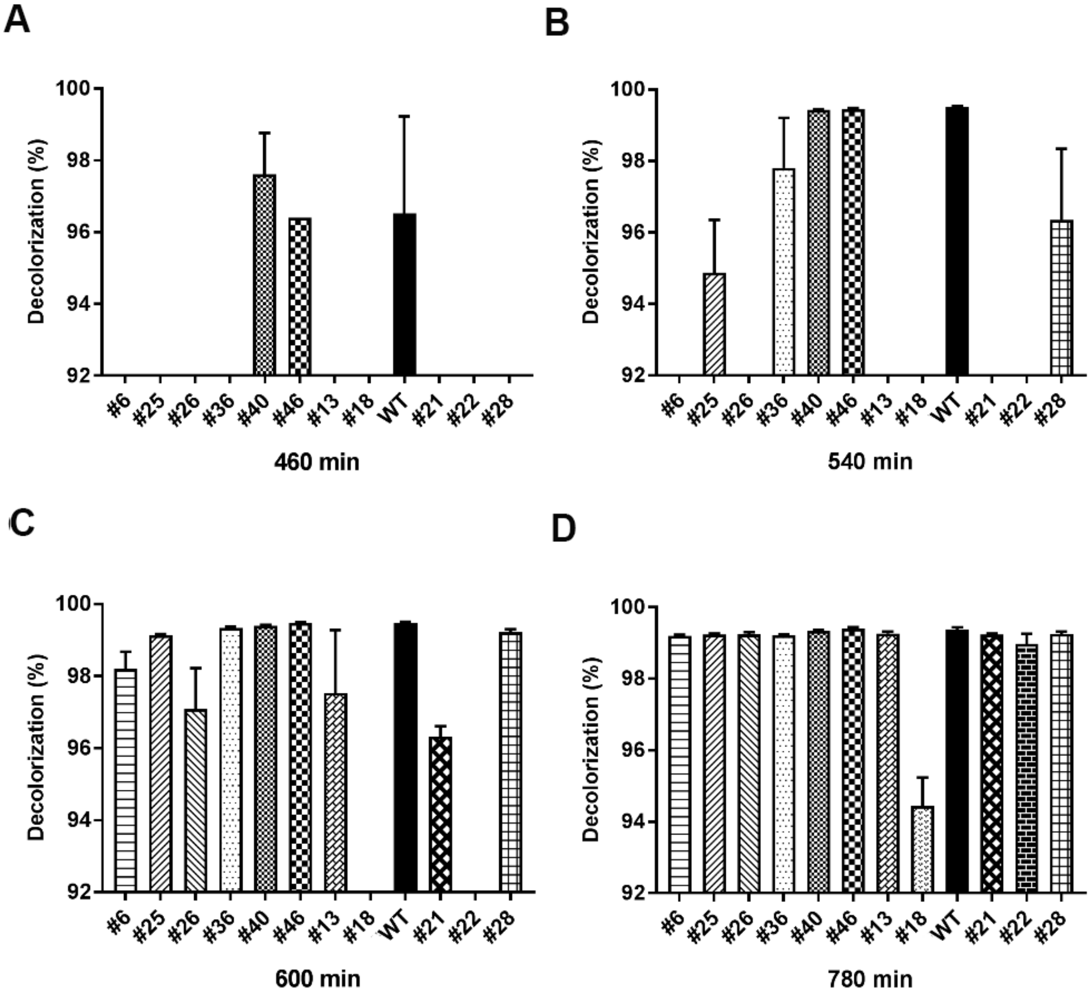

Figure S3. Aerobic degradation of AMR by *S. decolorationis* Ni1-3 and RNAP mutants. The initial concentration of AMR was 8 mM. Incubation conducted aerobically on 37 °C after 460 min (A), 540 min (B), 600 min (C) and 780 min (D).

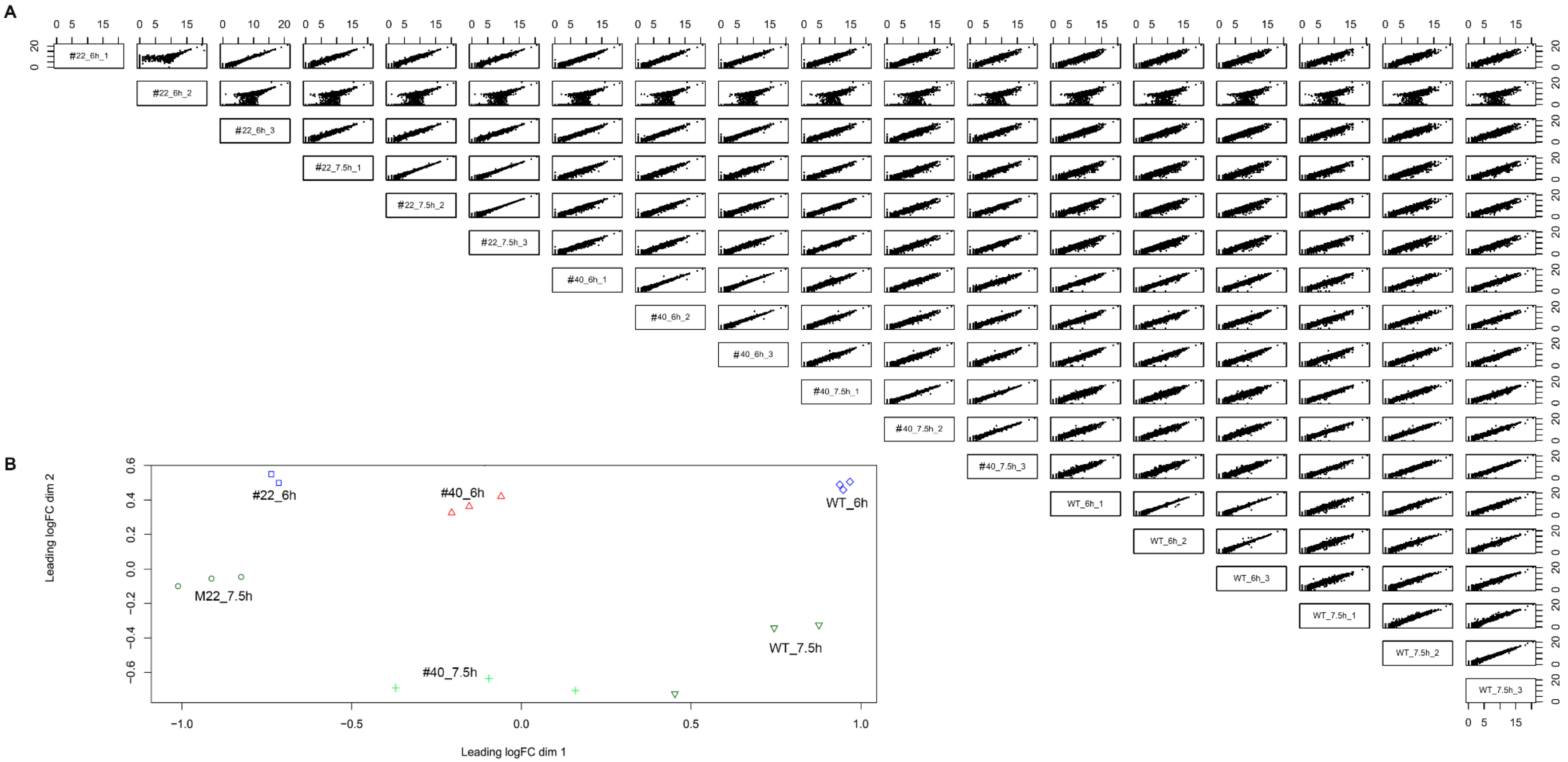

Figure S4. Pairwise scatter (A) and multidimensional scaling (MDS) (B) plot of the counts data.

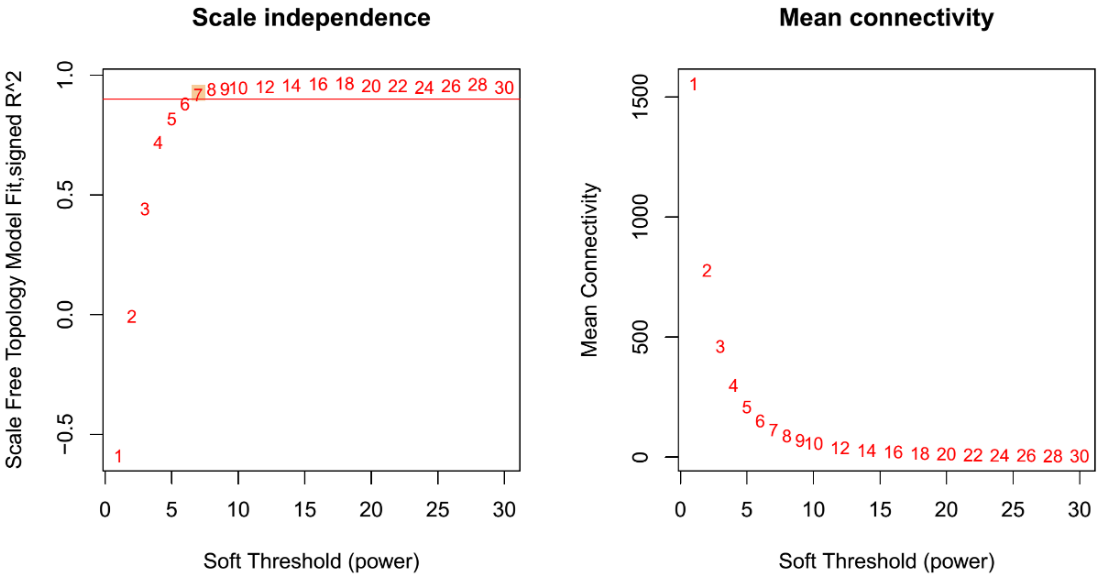

Figure S5. Soft-power pick for the construction of scale free network model.

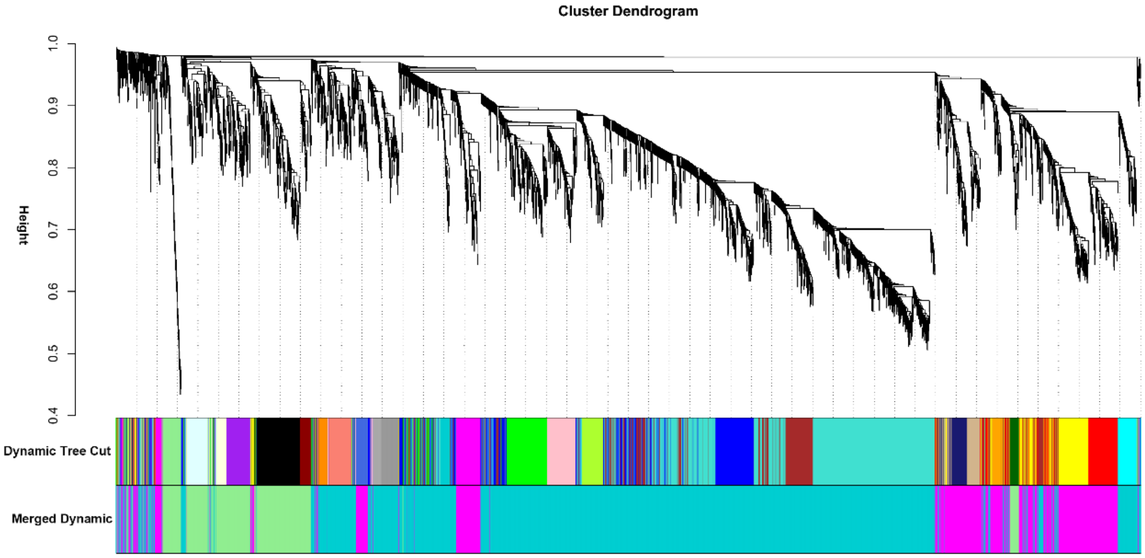

Figure S6. Merged dynamic tree of the gene module.

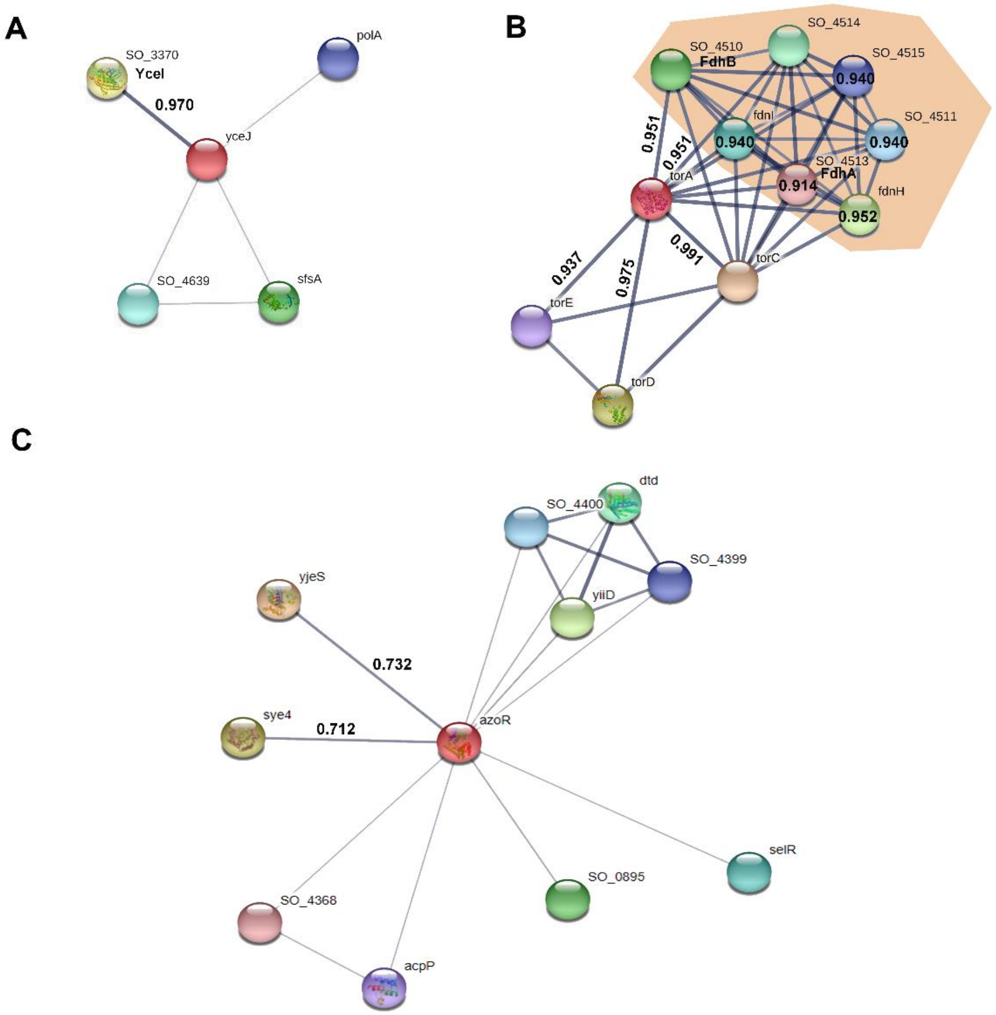

Figure S7. Analysis of protein-protein interaction networks using STRING. Network line thickness indicates the strength of data support. Combined score higher than 0.700 are shown in the figure. Network nodes marked with brown shadow are formate dehydrogenase subunits.

As TorA has been previously identified as a molybdenum enzyme, the activity of which could be inhibited by tungstate (Dawood and Azooz, 2019). After adding sodium tungstate to the degradation mixture, a great deficiency in AMR degradation was observed in the mutants and the WT strains (Fig. S8A). Meanwhile, alternation patterns of TMAO reduction efficiency between Ni1-3 and the mutants were identical to that of AMR degradation (Fig. S8B), which indirectly verified that TorA played important roles in AMR degradation. Although another molybdenum enzyme NapA exists in Ni1-3, it was observed not significantly correlated to AMR degradation with a GS of -0.40 (p = 0.116). However, TorA is a periplasmic protein, and AMR could not cross the cellular outer-membrane barrier, which implies that TorA may be an electron mediator component in the AMR degradation chain.

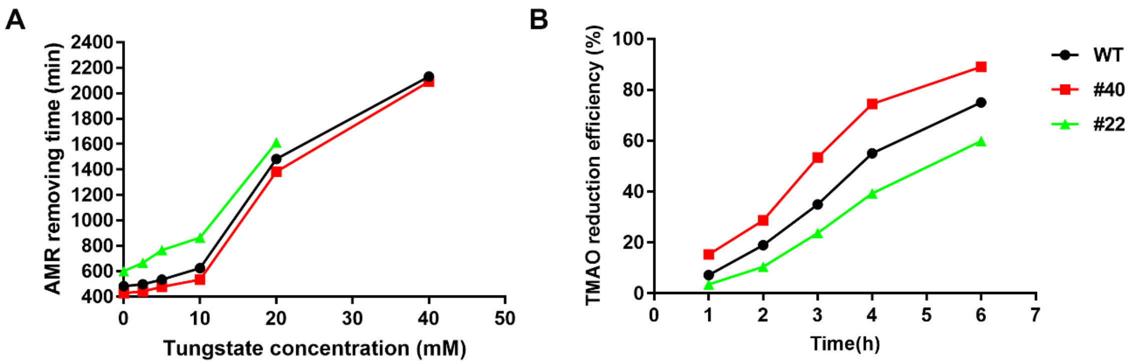

Figure S8. Phenotype verification of the key protein involvement in the suggested azo dye degradation pathway in *S. decolorationis*. (A). Inhibition of AMR degradation by adding sodium tungstate. (A). Comparison of the TMAO reduction efficiencies between the WT strain and the mutants.

Supplementary Materials and Methods

1.1 Seed Inoculum Preparation and Bacerial Growth Monitoring

The frozen WT strain at -80°C was thawed to inoculate into LB medium by the proportion of 10% (v/v) and routinely cultured as described in “2.1 Bacterial Strains and Growth Conditions” in the main text. After ten hours incubation, the optical density at 600 nm (OD600) were measured continuously per 30 min to determine the cell density in the culture. When OD600 reached 1.0, the bacterial culture was ready for inoculation as the seed inoculum. To monitor the bacterial growth, 2% (vol/vol) of the seed inoculum was mixed with 98% LB in a 96-well plate, incubated constantly at 37 °C for 36 h. The turbidity of the cell suspension was automatically measured via real-time OD600 monitoring in the microplate reader (Synergy HTX multi-mode reader, BioTek, USA) per 30 min.

1.2 PCR of the *rpoB* Gene

The DNA region corresponding to the *rpoB* gene of the mutants and WT strain was amplified by colony PCR. One primer pair was designed based on the genomic sequence of this region (she_ropb_m_f: 5’-GAACTAGTTGAAGCTGGTGCTTCAG-3’, she_ropb_m_r: 5’-CACCGTATTCTTCTAATGCGTCCAG-3’). Amplification was carried out using PrimeSTAR® HS kit (R010A, Takara, Japan) according to the instructions. PCR was processed in a 50 µL mixture containing 2 µL of each primer (10 µM), 25 µL 2 × PCR Master Mix (K0171, Thermo Scientific™, China), 19 µL sterilized water, 2 µL deionized water dissolved colony. The samples were initially denatured at 95 °C for 5 min, followed by 30 cycles of denaturation at 95 °C for 30 s, annealing at 58 °C for 30 s, extension at 72 °C for 4 min, following a final extension at 72 °C for 10 min.

1.3 RNA-seq

The total RNA was extracted by the Novogene company. RNA concentration was determined by using a NanoDrop™ 2000 Spectrophotometer (Thermo Fisher Scientific, USA), RNA quality was assessed using an Agilent2100 bioanalyzer (Agilent Technologies, Santa Clara, CA), and RNA samples with RNA integrity (RIN) > 8.0 were kept for the next steps. Ribosomal RNA was removed by using the RiboZero™ rRNA Removal Kit (Epicenter, Madison, USA). Strand specific cDNA libraries were constructed with 350 bp insert size and then sequenced using PE150 strategy on Illumina NovaSeq platform. Quality control of the raw reads was performed, including adapter trimming and low-quality reads (Phred score Q20 ≥ 95%) removal.

1.4 Verification of the TorA Involvement in Azo Dye Degradation

As AMR degradation was found correlating to a novel non-CymA-Mtr pathway in this study, of which TorA (a molybdenum enzyme, identified as a TMAO reductase (Mintmier et al., 2020)) was the key enzyme. To verify the correlation between molybdenum enzyme activity and AMR degradation activity, gradient concentrations of sodium tungstate (0, 2.5 mM, 5 mM, 10 mM, 20 mM and 40 mM) were added in the AMR degradation mixture of each strain (i.e., #40, #22 and WT) to inhibit the molybdenum enzyme activity (Dawood and Azooz, 2019), and the AMR degradation activity was measured simultaneously. To check the TMAO reduction activity, TMAO substituted for AMR and added into the

modified MR2A medium as the sole electron acceptor in a final concentration of 8 g/L under anaerobic conditions, and sampling was conducted at 1 h, 2 h, 3 h, 4 h and 6 h. Sample preparation and TMAO reduction activity measurement were conducted according to the manufacture's instruction of JingMei TMAO-ELISA Kit (JM-07766F2, JingMei Biotechnology, China).

References

Dawood, M.; Azooz, M. (2019). Concentration-dependent effects of tungstate on germination, growth, lignification-related enzymes, antioxidants, and reactive oxygen species in broccoli (*Brassica oleracea* var. *italica* L.). *Environ. Sci. Pollut. Res. 26*, (36), 36441-36457.

Mintmier, B.; Nassif, S.; Stolz, J. F.; Basu, P. (2020) Functional mononuclear molybdenum enzymes: challenges and triumphs in molecular cloning, expression, and isolation. *J. Biol. Inorg. Chem. 25*, (4), 547-569.
